# Supplementary material for: Evidence of acclimatization or adaptation in Hawaiian corals to higher ocean temperatures
Source: PeerJ. 2018 Aug 7;6:e5347. doi: 10.7717/peerj.5347 (PMC6086081; doi:10.7717/peerj.5347)
Supplement: Figure S1 — Visual assessment of tested coral species throughout the stress (11 July–11 Aug 2017) and recovery periods. Scale (1, normal; 2, pale; 3, bleached; 4, dead). [file peerj-06-5347-s002.pdf]

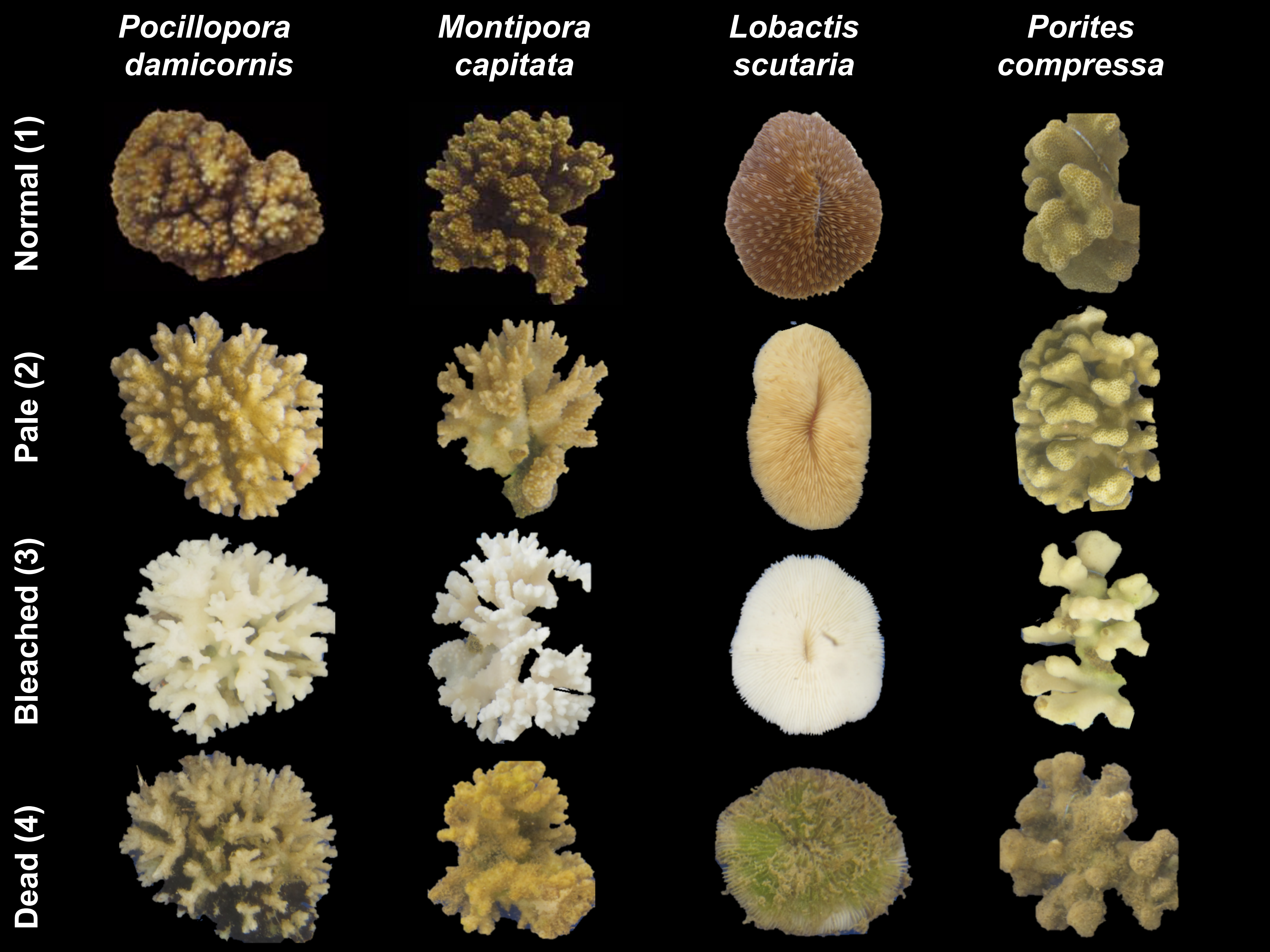

Pocillopora damicornis
Montipora capitata
Lobactis scutaria
Porites compressa
Normal (1)
Pale (2)
Bleached (3)
Dead (4)
