# Supplementary material for: Evidence of acclimatization or adaptation in Hawaiian corals to higher ocean temperatures
Source: PeerJ. 2018 Aug 7;6:e5347. doi: 10.7717/peerj.5347 (PMC6086081; doi:10.7717/peerj.5347)
Supplement: Table S1 — Cox proportional hazards regression analysis, with censoring of individuals that survived to the end of the experiment. Coral time to mortality was recorded as the number of days since the start of the experiment within each year. [file peerj-06-5347-s003.docx]

**Table S1.** Cox proportional hazards regression analysis, with censoring of individuals that survived to the end of the experiment. Coral time to mortality was recorded as the number of days since the start of the experiment within each year.

|  | **Proportional Mortality Analysis** | | | | | | |
| --- | --- | --- | --- | --- | --- | --- | --- |
|  |  | Cox Proportional Hazards with Censoring | | | |  |  |
|  |  | **Risk Ratio** | **p** | **Parameter** | **Estimate** | **SE** | **n** |
| ***Fungia scutaria*** | ***2017 - 1970*** | 0.052 | **<0.0001** | Year [1970] | 1.482 | 0.393 | 26 |
|  | ***1970 - 2017*** | 19.376 | **<0.0001** |  |  |  |  |
|  |  |  |  |  |  |  |  |
| ***Montipora capitata*** | ***2017 - 1970*** | 0.0785 | **<0.0001** | Year [1970] | 1.272 | 0.234 | 55 |
|  | ***1970 - 2017*** | 12.739 | **<0.0001** |  |  |  |  |
|  |  |  |  |  |  |  |  |
| ***Pocillopora***  ***damicornis*** | ***2017 - 1970*** | 0.298 | **0.0003** | Year [1970] | 0.605 | 0.154 | 61 |
|  | ***1970 - 2017*** | 3.351 | **0.0003** |  |  |  |  |
